# Using genome-scale metabolic models to compare serovars of the foodborne pathogen *Listeria monocytogenes*

**DOI:** 10.1371/journal.pone.0198584

**Published:** 2018-06-07

**Authors:** Zachary P. Metz, Tong Ding, David J. Baumler

**Affiliations:** 1 Department of Food Science and Nutrition, University of Minnesota-Twin Cities, St. Paul, Minnesota, United States of America; 2 Microbial and Plant Genome Institute, University of Minnesota-Twin Cities, St. Paul, Minnesota, United States of America; 3 Biotechnology Institute, University of Minnesota-Twin Cities, St. Paul, Minnesota, United States of America; University of Wisconsin Madison, UNITED STATES

## Abstract

*Listeria monocytogenes* is a microorganism of great concern for the food industry and the cause of human foodborne disease. Therefore, novel methods of control are needed, and systems biology is one such approach to identify them. Using a combination of computational techniques and laboratory methods, genome-scale metabolic models (GEMs) can be created, validated, and used to simulate growth environments and discern metabolic capabilities of microbes of interest, including *L*. *monocytogenes*. The objective of the work presented here was to generate GEMs for six different strains of *L*. *monocytogenes*, and to both qualitatively and quantitatively validate these GEMs with experimental data to examine the diversity of metabolic capabilities of numerous strains from the three different serovar groups most associated with foodborne outbreaks and human disease. Following qualitative validation, 57 of the 95 carbon sources tested experimentally were present in the GEMs, and; therefore, these were the compounds from which comparisons could be drawn. Of these 57 compounds, agreement between *in silico* predictions and *in vitro* results for carbon source utilization ranged from 80.7% to 91.2% between strains. Nutrient utilization agreement between *in silico* predictions and *in vitro* results were also conducted for numerous nitrogen, phosphorous, and sulfur sources. Additionally, quantitative validation showed that the *L*. *monocytogenes* GEMs were able to generate *in silico* predictions for growth rate and growth yield that were strongly and significantly (p < 0.0013 and p < 0.0015, respectively) correlated with experimental results. These findings are significant because they show that these GEMs for *L*. *monocytogenes* are comparable to published GEMs of other organisms for agreement between *in silico* predictions and *in vitro* results. Therefore, as with the other GEMs, namely those for *Escherichia coli*, *Staphylococcus aureus*, *Vibrio vulnificus*, and *Salmonella* spp., they can be used to determine new methods of growth control and disease treatment.

## Introduction

Due to the importance of *L*. *monocytogenes* to the food industry, there is a continuous interest in new methods of control and treatment. The development of rapid, relatively cheap genome sequencing techniques in recent years has led to the emergence of genomic tools for identifying more properties of microorganisms through the field of systems biology and its associated computational techniques.

Genome-scale metabolic models (GEMs) are one of the newer techniques by which foodborne pathogens are being studied. These GEMs take the genetic information contained in the entire genome and convert it to a metabolic network that consists of metabolic reactions and their associated metabolites. This network is then converted to a system of algebraic equations. Using computing software, this system of equations can be used to calculate the flow of metabolites through the metabolic network and predict the growth of the organism under specific conditions. The model can then be adjusted by comparing the predictions to experimental data. A working model can then be used to study the metabolism of the organism and identify metabolic reactions that are essential for the growth and survival of the organism. These essential reactions provide ideal targets for new methods of treatment and control. This type of approach, using GEMs to study metabolism and identify new targets for control of foodborne pathogens, has already been done for several foodborne pathogens, including *Escherichia coli* O157:H7, *Salmonella*, *Vibrio vulnificus*, *Staphylococcus aureus*, *Listeria monocytogenes* [1,2,3,4,5,6,7,8,9,10]. However, many genera of foodborne pathogens have not been examined beyond a single strain through the use of computational modeling using GEMs.

The work presented here investigates the validity of using semi-automated online tool KBase to construct the draft GEM, which can be done in a span of hours [The Department of Energy Systems Biology Knowledgebase (KBase). Available online at: https://kbase.us]. The semi-automated process uses publicly available genomic information alongside online database tools to generate draft GEMs. The draft GEMs are then curated and expanded through an iterative process to validate computational predictions by comparison to experimental data. GEMs generated through use of KBase have been used to elucidate the metabolic profile of *Candidatus* Homothermaceae, to reveal niche adaptation of *Sinorhizobium meliloti*, to reveal diverse metabolic functions used in plant-bacterial interactions of *Pseudomonas fluorescens*, and to analyze central metabolism across all microbial life through analysis of > 8,000 different prokaryotic organisms [11,12,13,14].

The usefulness of computational models of microorganisms increases through validation with experimental data. GEMs are commonly qualitatively validated through nutrient utilization data for carbon, nitrogen, phosphorous, and sulfur to iteratively improve the accuracy of *in silico* growth predictions. To increase the accuracy of the *in silico* predictions beyond the improvements made through qualitative validation, it is necessary to quantitatively validate the GEMs with additional experimental data.

Quantitative validation is primarily accomplished by performing aerobic batch growth experiments that yield growth rates (h^-1^) and biomass yields (gDCW/g glucose) as previously described [1]. These experimental values can be compared to *in silico* predictions, and allow for adjustments to the model which improve its predictive power and accuracy for quantitative growth measurements. The improved GEMs can then be used to predict how *L*. *monocytogenes* will behave under a wide variety of different conditions prior to performing rigorous laboratory techniques. Additionally, this data can be used to generate numerical values that can convert between experimentally determined data, such as viable cell counts, optical density, and biomass. These conversion factors can be used to utilize existing *L*. *monocytogenes* growth data for new *in silico* inquiry using GEMs.

Here we describe the use of semi-automated GEMs for studying six strains of *L*. *monocytogenes*, and also comparison to prior study that used a condensed GEM for *L*. *monocytogenes* strain EGD-e to investigate listerial metabolism during intracellular replication used in the course of invasion of a human cell to cause disease [10]. The six *L*. *monocytogenes* strains chosen for this study all come from one of the three serovars most frequently responsible for human listeriosis cases: 1/2a, 1/2b, and 4b. Strains J2-031 and JO161 come from serovar 1/2a, which belongs to lineage II. Strains J2-064 and R2-502 belong to serovar 1/2b and lineage I. Finally, Strains F2365 and ScottA belong to serovar 4b and lineage I. These six GEMs, validated through comparison to experimental data, will identify new target areas for future control mechanisms in foods and food processing environments. Additionally, the GEMs will provide a fresh perspective on the metabolism of *L*. *monocytogenes* at a comparative genome level through use of GEMs.

## Results

A numerical comparison of the genomes of the six strains was accomplished using the RAST (Rapid Annotation using Subsystem Technology) database (Table 1) [15]. Each strain has approximately 3,000 genes, with 85%– 90% of those genes shared among all of the strains. Less than 3% of the genes for each strain are unique, and none of the three serovars chosen had more than 53 genes unique to that serovar.

**Table 1:** Numerical genome comparison of the six chosen strains of *L*. *monocytogenes*.

*L*. *monocytogenes* strain (ILSI #)	Source	Genes (% Shared)	Unique Genes (% of Genome)	Serovar	Serovar Specific Genes
J2-031	Animal isolate (bovine) 1996	3,009 (86.6%)	49 (1.63%)	1/2a	53
JO161	Human epidemic 2000	3,024 (86.1%)	65 (2.15%)		
J2-064	Food epidemic 1994 (Illinois)	2,938 (88.7%)	16 (0.54%)	1/2b	13
R2-502	Animal isolate (bovine) 1989	3,069 (84.9%)	57 (1.86%)		
F2365	Food epidemic 1985 (L.A)	2,907 (89.6%)	32 (1.10%)	4b	26
ScottA	Human epidemic 1993	3,016 (86.4%)	73 (2.42%)		

### Reconstruction of genome-scale metabolic models

The number of genes, metabolites, and metabolic reactions present in GEMs are important characteristics (Table 1). The created GEMs contained just under 800 genes for each strain (slightly more than 25% of the ORFs contained in each genome). Each GEM also contained over 1,100 metabolites, and a cumulative total of 1,116 metabolites were contained in the six GEMs. 1,106 of these metabolites were shared between all six of the *L*. *monocytogenes* strains. In the work of Schauer et al. (2010), a condensed central metabolic network was reconstructed to model listerial metabolism, and the GEM contained 155 reactions (30 are transport reactions) and 167 metabolites [10]. Many of the reactions and metabolites contained in the condensed GEM for *L*. *monocytogenes* strain EGD-e are contained in the GEMs constructed through K-Base as part of this work, but the GEMs reconstructed here contain >1,000 additional reactions and >900 metabolites due to extensive micronutrients contained in the detailed biomass equation, and also through new reaction additions through comparison to experimental data. There was also one metabolite unique to *L*. *monocytogenes* strain J2-064 (p-Hydroxybenzaldehyde), and one metabolite unique to serovar 1/2a (Toxopyrimidine). Draft GEMs contained over 1,050 reactions. Surveying the metabolites contained in the stoichiometric matrix of each of the GEMs for additional carbon sources added almost 200 reactions, and the final curation added approximately 100 reactions to each GEM. The cumulative number of reactions contained in all six GEMs was 1,335, and 1,300 of these reactions were shared among all six GEMs (Table 2). Of the 1,335 possible reactions, one was unique to *L*. *monocytogenes* strain F2365, seven were unique to *L*. *monocytogenes* strain J2-031, two were unique to *L*. *monocytogenes* strain J2-064, and one reaction was unique to serovar 1/2a (Table 3). No unique reactions were found in the GEMs for *L*. *monocytogenes* strains ScottA or R2-502.

**Table 2:** Gene, metabolite, and reactions contained in each version of the GEMs.

Strain	Genes (% of Genome)	Metabolites	Draft GEM Reactions	Reactions After Validation with Carbon Data	Final Reactions
J2-031	770 (25.6%)	1,112	1,053	1,219	1,320
JO161	786 (26.0%)	1,115	1,055	1,221	1,320
J2-064	783 (26.7%)	1,114	1,056	1,221	1,320
R2-502	786 (25.6%)	1,114	1,057	1,223	1,322
F2365	780 (26.8%)	1,110	1,053	1,219	1,318
ScottA	779 (25.8%)	1,110	1,053	1,219	1,318

**Table 3:** Enzymes catalyzing strain and serovar-specific metabolic reactions.

Strain (Serovar)	Unique Enzymes
J2-031 (1/2a)	S-Adenosyl-L-homocysteine hydrolase rxn00257_c0 Isocitrate glyoxylate-lyase, alpha-D-Glucose-1-phosphate:alpha-D-glucose-1-phosphate Xanthosine-5’-phosphate:L-glutamine amido-ligase (AMP-forming) rxn01615_c0 4-amino-5-hydroxymethyl-2-methylpyrimidine synthetase_c0
J2-064 (1/2b)	4-hydroxybenzaldehyde:NAD+ oxidoreductase 4-hydroxy-benzyl-alcohol dehydrogenase
F2365 (4b)	Adenosyl cobinamide kinase
J2-031 and JO161 (1/2a)	ATP:4-amino-5-hydroxymethyl-2-methylpyrimidine 5-phosphotransferase_c0

### Nutrient phenotype data

Also of interest is the degree to which the different strains and different serovars of *L*. *monocytogenes* differ in their nutrient utilization. Biolog^™^ PM plates are widely used to quickly generate large quantities of nutrient utilization data that can be used to qualitatively validate GEMs. Upon completion of the Biolog^™^ PM experiments, strain and serovar specific nutrient utilization data became available for these six *L*. *monocytogenes* strains. 95 sources of carbon, 95 sources of nitrogen, 59 sources of phosphorous, and 35 sources of sulfur were analyzed, and the number of compounds capable of serving as the sole source of the respective nutrients was determined (S2 Dataset). The strain-specific and serovar-specific differences in nutrient utilization were also examined for the same four nutrients (Table 4), and the unique nutrients for each strain and serovar were identified (Table 5).

**Table 4:** Number of strain and serovar specific nutrients.

	Carbon Sources	Nitrogen Sources	Sulfur + Phosphorus Sources
All strains	11	7	3
No strains	24	82	87
Unique to J2-031	3	0	0
Unique to JO161	4	0	0
Unique to J2-064	1	0	0
Unique to R2-502	3	0	1
Unique to F2365	4	0	0
Unique to ScottA	0	1	0
Unique to 1/2b	1	1	2

**Table 5:** Unique nutrient sources metabolized by a single *L*. *monocytogenes* strain or serovar.

**Strain/Serovar**	**Unique Carbon Sources**
J2-031	Formic Acid, D-Aspartic Acid, M-Tartaric Acid
JO161	D-Melibiose, D-Threonine, Glyoxylic Acid, L-Serine
J2-064	Glycyl-L-Proline
R2-502	Adonitol, M-Inositol, L-Lyxose
F2365	Sucrose, α-Hydroxy Glutaric Acid-γ-Lactone, Fumaric Acid, Tyramine
1/2b	p-Hydroxy Phenyl Acetic Acid
**Strain/Serovar**	**Unique Nitrogen Sources**
ScottA	L-Threonine
1/2b	D-Valine
**Strain/Serovar**	**Unique Phosphorus/Sulfur Sources**
R2-502	2-Hydroxyethane Sulfonic Acid
1/2b	Thiosulfate, Tetrathionate

#### Comparison between *in silico* predictions and experimental nutrient utilization results

The previously described nutrient phenotype data can be compared to *in silico* predictions as part of the qualitative validation process. These comparisons allow the reconciliation of inaccuracies of *in silico* predictions, which results in optimized, validated GEMs. When comparing *in silico* predictions to experimental results for utilization of nutrients, there can be two types of disagreement: either the model predicts growth, but experimentally there was none (termed “false positive”), or the model predicts no growth, but experimentally there was growth (termed “false negative”).

**Fig 1:**
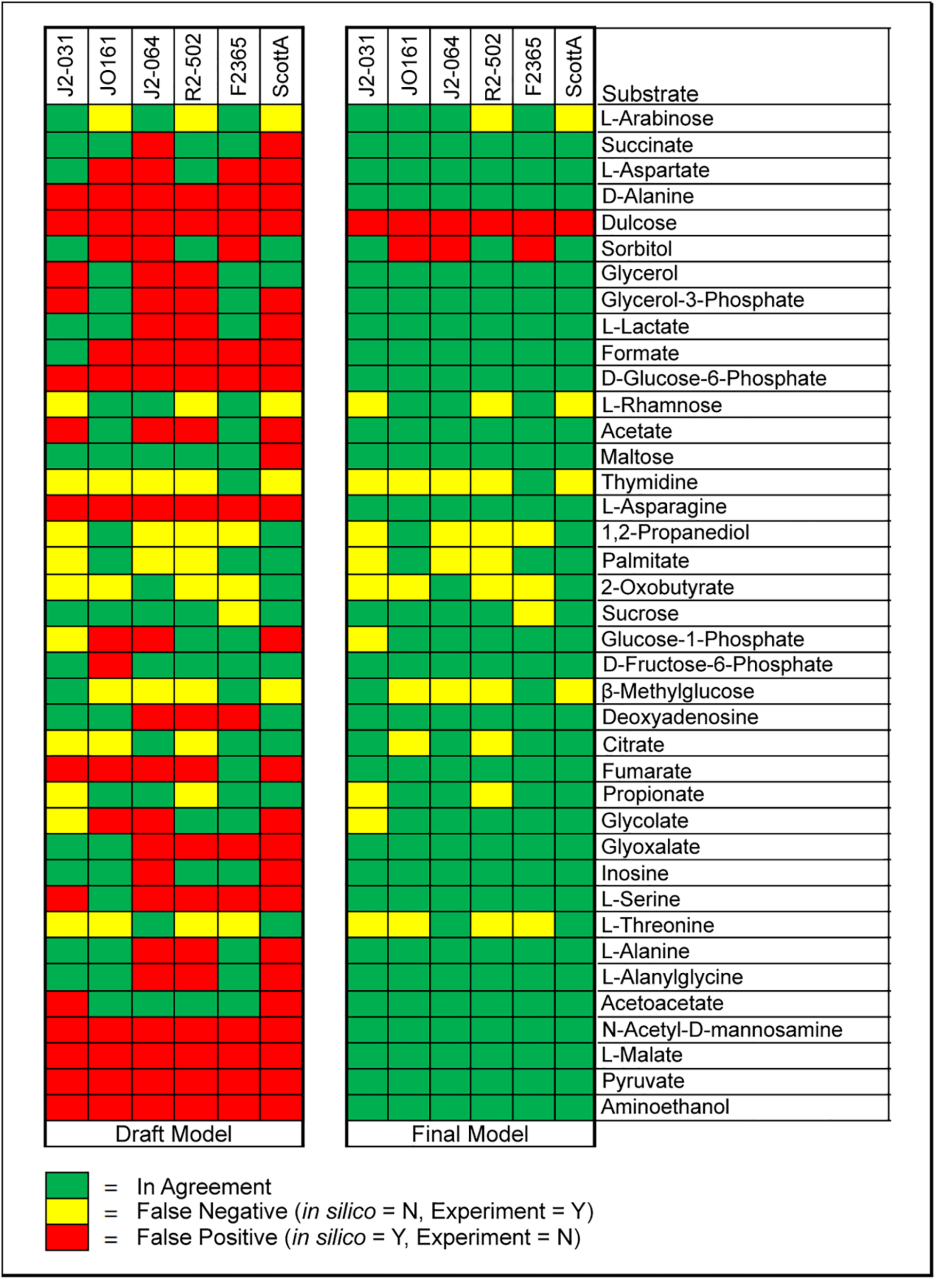
Comparison of *in silico* predictions to experimental results for 39 individual carbon sources. The 39 carbon sources displayed are those with at least one disagreement.

A comparison of agreement between draft and final models for carbon source utilization shows a drastic improvement in agreement following gapfilling and manual curation (Fig 1). The draft model contains the initial nutrient utilization predictions, while the final model contains predictions generated following manual curation and gapfilling. 57 carbon sources found in the PM1 plates were also present in the GEMs, allowing comparison between *in silico* predictions and experimental data for utilization of these nutrients, and of these glucose, fucose, glycerol, acetate, and citrate were investigated with the condensed *L*. *monocytogenes* GEM in previous work from Schauer et al. [10]. In this study, 18 of these carbon sources (N-acetyl-D-glucosamine, L-proline, D-trehalose, D-mannose, D-serine, D-gluconic acid, D-mannitol, L-glutamate, D-fructose, D-glucose, uridine, L-glutamine, amylotriose, adenosine, glycyl-L-aspartate, D-cellobiose, glycyl-L-glutamate, and glycyl-L-proline) resulted in agreement between *in silico* predictions and experimental results for all strains. It was found that draft GEMs agreements ranged from 48.3% to 69.0%. Following manual curation, the final GEMs, agreements ranged from 80.7% to 91.2%, and each strain had a minimum of four false negatives and one false positive (Fig 1).

**Fig 2:**
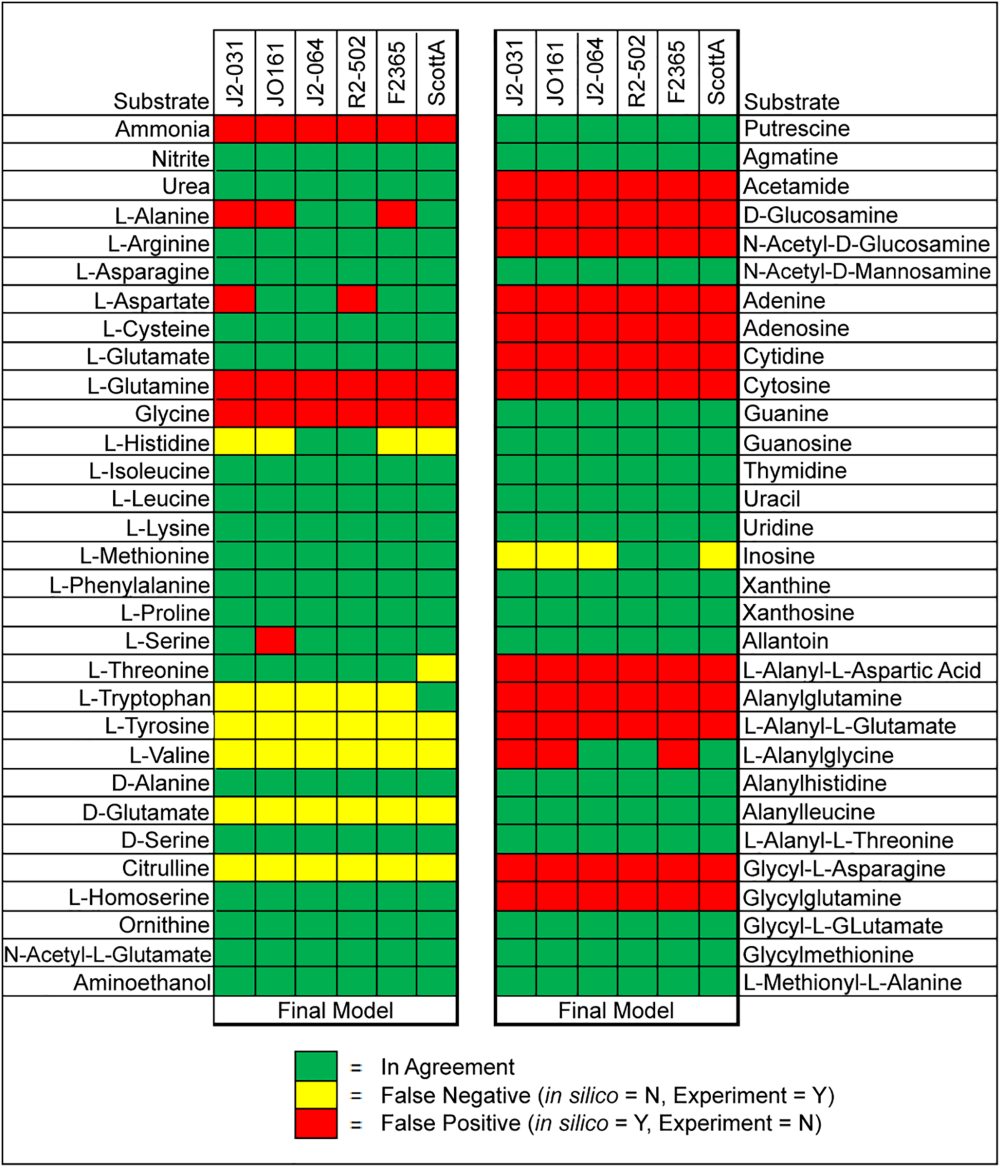
Comparison of *in silico* predictions to experimental results for 62 individual nitrogen sources.

After manual curation and gapfilling to improve the agreement between carbon utilization experimental data and the corresponding *in silico* predictions, a similar comparison was made for nitrogen (Fig 2). 62 nitrogen sources found in the PM3 plates were also present in the models, allowing comparisons to be made. It was found that agreement ranged from 59.7% to 66.1%, and all strains had at least five false negatives and at least 15 false positives (Fig 2). In contrast to carbon, GEMs were not manually curated to improve agreement for nitrogen, phosphorous, or sulfur sources since the genes and reactions for many of metabolic pathways for many of these nutrients is not known for *L*. *monocytogenes*, and may be addressed in future studies to improve and update these GEMs for more accurate utilization of these nutrients.

**Fig 3:**
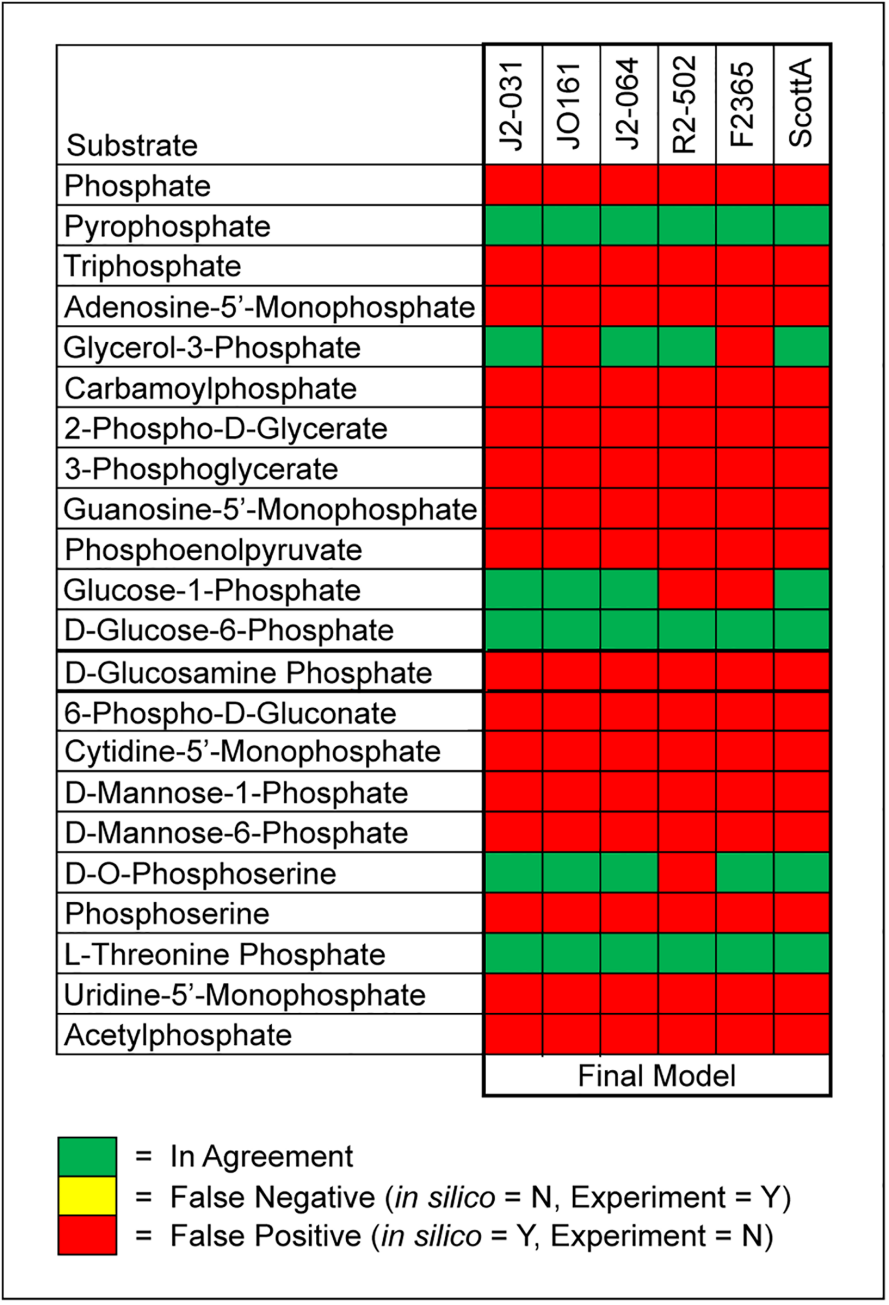
Comparison of *in silico* predictions to experimental results for 22 individual phosphorus sources.

**Fig 4:**
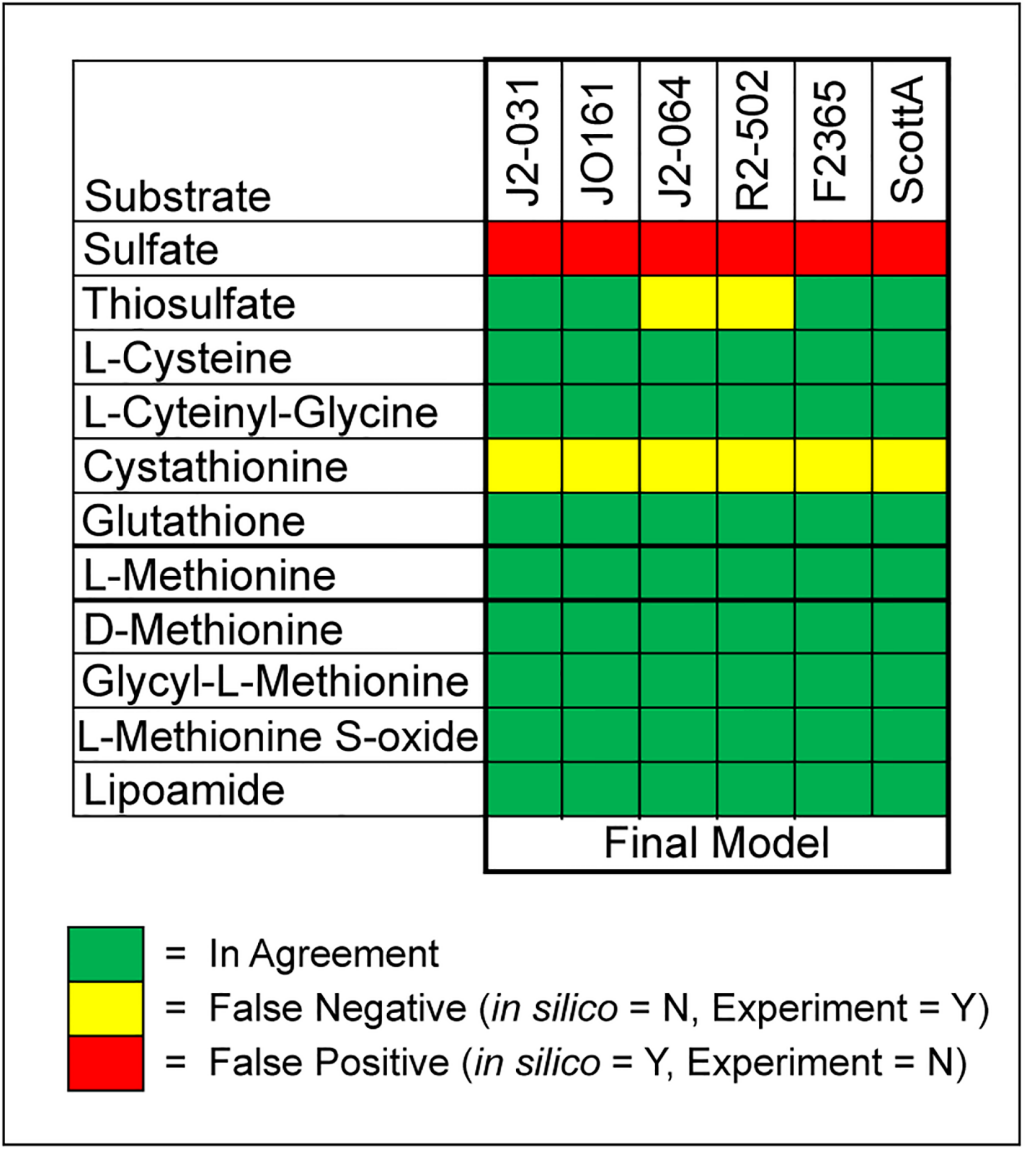
Comparison of *in silico* predictions to experimental results for 11 sulfur sources.

Comparisons between *in silico* predictions and experimental results for phosphorous and sulfur utilization were made in conjunction with the nitrogen comparisons (Figs 3 and 4). For phosphorous, 22 compounds enabled comparisons. For these 22 compounds, agreement ranged from 18.2% to 27.3%, and each strain had at least 16 false positives. However, none of the strains had any false negatives (Fig 3). For sulfur 11 compounds were found in both the PM4 plates and the GEMs. For these 11 compounds, agreement ranged from 72.7% to 81.8%, and each strain had at least one false negative and one false positive (Fig 4).

#### Agreement comparison to other genome-scale metabolic models

**Fig 5:**
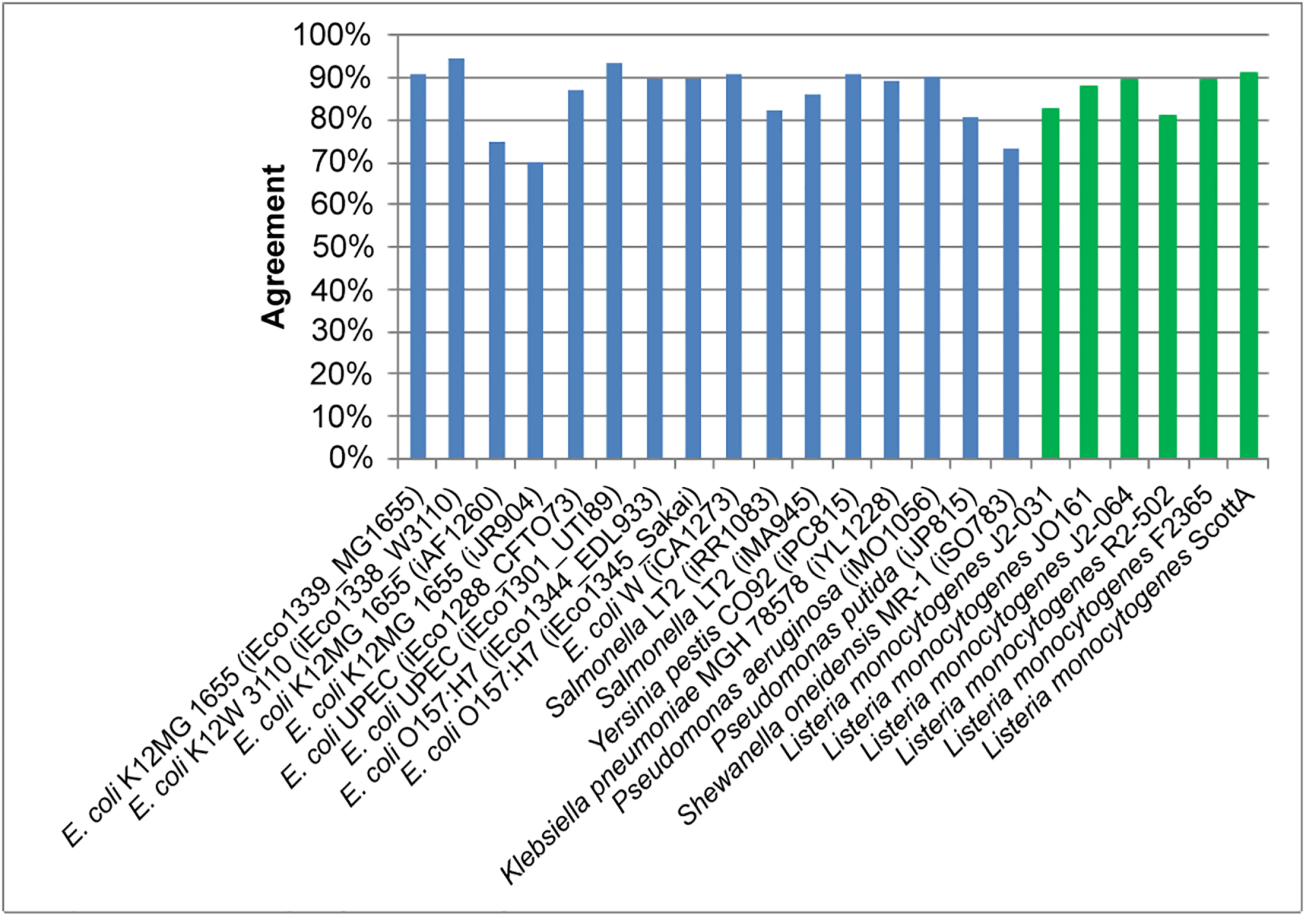
Comparison of carbon source utilization agreement between this study and previous studies. The carbon source utilization agreements of the six genome-scale metabolic models created in this study (green) are compared to the same agreements in 16 previously created models (blue).

**Fig 6:**
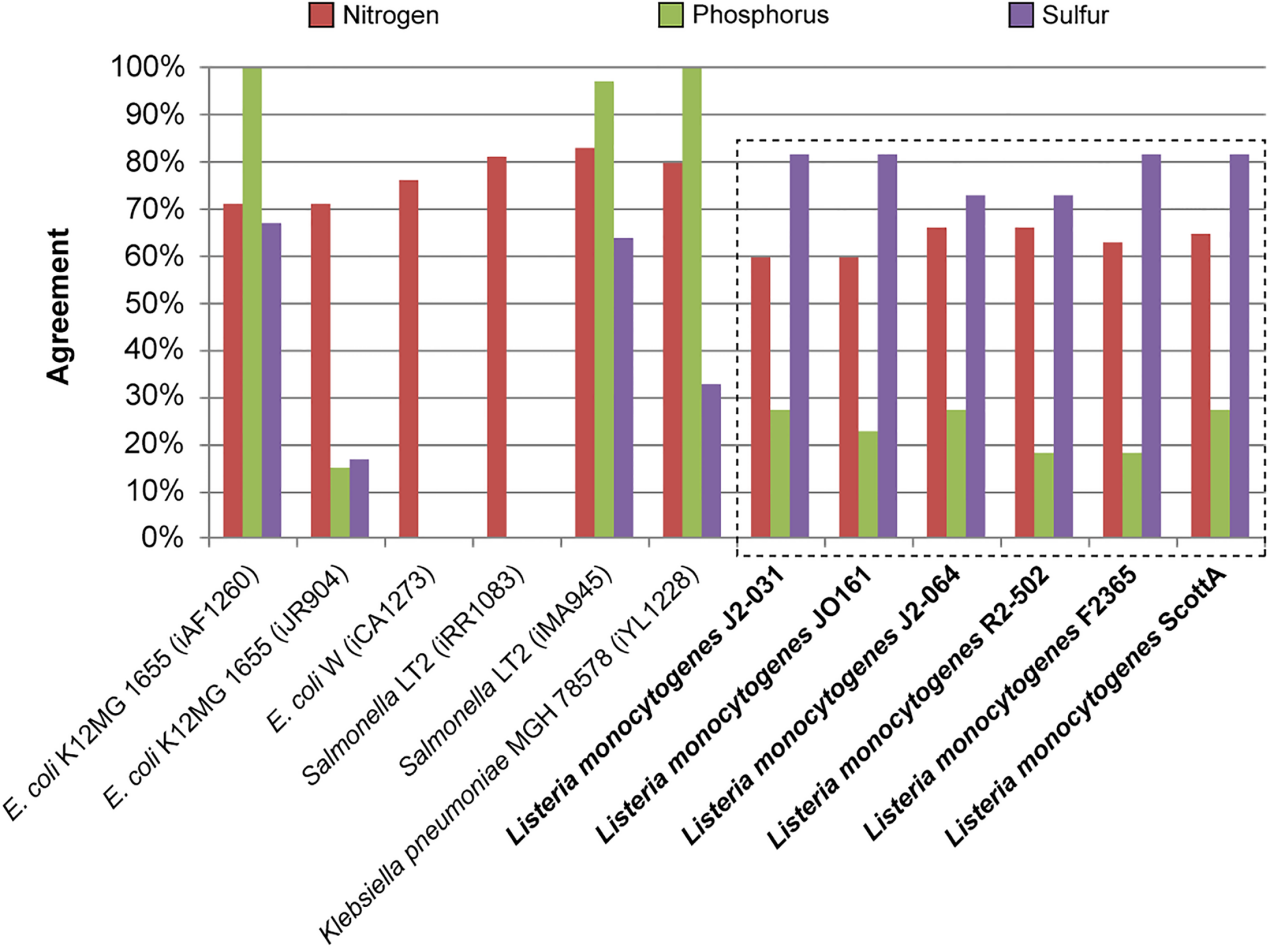
Comparison of nitrogen, phosphorus, and sulfur utilization agreement between this study and previous studies. The nutrient utilization agreements of the six genome-scale metabolic models created in this study (dashed box) are compared to the same agreements in six previously created models for nitrogen (red), and four for phosphorus (green) and sulfur (purple).

It is worthwhile to compare the GEMs generated by this work to those previously created for other organisms in order to assess the validity of the semi-automated model creation approach. In the work of Schauer et al. (2010), the condensed GEM for *L*. *monocytogenes* strain EGD-e was used to investigate network robustness and predict extreme pathways and elemental nodes for metabolism of glucose, fucose, glycerol, acetate, and citrate as carbon sources, but no experimental validation for utilization of these carbon sources was conducted [10]. Of the six *L*. *monocytogenes* strains chosen in this this study that had GEMs reconstructed and updated through comparison to nutrient utilization experiments, revealed more strain to strain variability in utilization of glucose (6/6), fucose (5/6), glycerol (3/6), acetate (2/6), citrate (3/6) as a sole source of carbon. The carbon agreement of the six newly created *L*. *monocytogenes* GEMs was compared to the carbon agreement (determined through use of Biolog phenotypic arrays) of 16 previously published GEMs (Fig 5), nitrogen agreement was compared to six previously published GEMs (Fig 6), and phosphorus and sulfur agreements were compared to four previously published GEMs (Fig 6) [1,3,16,17,18,19,20,21,22,23].

### Essential reactions

Finally, the number of reactions essential to the survivability and viability of *L*. *monocytogenes* were predicted in simulations reflecting glucose minimal media as well as five high risk foods (Table 6). The five high risk foods simulated were queso fresco, chicken breast, smoked salmon, cantaloupe, and romaine lettuce. There were approximately 370 predicted essential reactions when glucose minimal media was simulated, and this number decreased to approximately 300 in the high risk food simulations. Additionally, at least one unique essential reaction was predicted and identified in every simulation for *L*. *monocytogenes* strains J2-031, J2-064, and F2365, as well as serovar 1/2a (Tables 6 and 7). In the work of Schauer et al. the data from a combination of experimental genome-scale screening, and *in silico* extreme pathway and elementary mode analysis using a condensed GEM of *L*. *monocytogenes* strain EGD-e demonstrated a critical role of glycerol, fucose, and purine metabolism and synthesis of glutathione, aspartate semialdehyde, serine and amino acids during intracellular replication in human cells [10]. When compared to the essential reaction predictions for *L*. *monocytogenes* GEMs in food environments from this study, some essential reactions utilizing glycerol or other glycerol containing metabolites, purines, and aspartate semialdehyde were also identified, thus indicating a critical role for cellular replication in both foods and intracellular replication in human cells. In comparison to the nutrient limited conditions encountered by *L*. *monocytogenes* during intracellular replication, food environments are more nutrient rich environments containing all of the amino acids, and therefore the essential reactions for amino acid biosynthesis for *L*. *monocytogenes* GEMs in foods was not observed. Future studies with the GEMs constructed from this current study to determine the effects of gene mutations and corresponding predicted essential reactions may lead to new control targets for *L*. *monocytogenes* in foods.

**Table 6:** Essential reaction summary for the simulation of conditions representing glucose minimal media, queso fresco, chicken breast, smoked salmon, cantaloupe, and romaine lettuce.

Glucose Minimal Media				
Strain	Total (% Shared)	Unique (%)	Serovar	Serovar Specific Reactions
J2-031	369 (95.1%)	7 (1.90%)	1/2a	3
JO161	371 (94.6%)	0 (0%)		
J2-064	368 (95.4%)	2 (0.54%)	1/2b	0
R2-502	368 (95.4%)	0 (0%)		
F2365	367 (94.6%)	1 (0.27%)	4b	0
ScottA	367 (95.6%)	0 (0%)		
**Queso Fresco**				
J2-031	297 (93.6%)	9 (3.03%)	1/2a	1
JO161	295 (94.2%)	0 (0%)		
J2-064	306 (90.8%)	7 (2.29%)	1/2b	0
R2-502	294 (94.6%)	0 (0%)		
F2365	293 (94.9%)	1 (0.34%)	4b	0
ScottA	293 (94.9%)	0 (0%)		
**Chicken Breast**				
J2-031	297 (94.3%)	7 (2.36%)	1/2a	1
JO161	298 (94.0%)	0 (0%)		
J2-064	309 (90.6%)	7 (2.27%)	1/2b	0
R2-502	297 (94.3%)	0 (0%)		
F2365	296 (94.6%)	1 (0.34%)	4b	0
ScottA	296 (94.6%)	0 (0%)		
**Smoked Salmon**				
J2-031	297 (94.3%)	7 (2.36%)	1/2a	1
JO161	298 (94.0%)	0 (0%)		
J2-064	309 (90.6%)	7 (2.27%)	1/2b	0
R2-502	297 (94.3%)	0 (0%)		
F2365	296 (94.6%)	1 (0.34%)	4b	0
ScottA	296 (94.6%)	0 (0%)		
**Cantaloupe**				
J2-031	295 (94.9%)	5 (1.69%)	1/2a	1
JO161	298 (94.0%)	0 (0%)		
J2-064	309 (90.6%)	7 (2.27%)	1/2b	0
R2-502	297 (94.3%)	0 (0%)		
F2365	296 (94.6%)	1 (0.34%)	4b	0
ScottA	296 (94.6%)	0 (0%)		
**Romaine Lettuce**				
J2-031	297 (94.3%)	7 (2.36%)	1/2a	1
JO161	298 (94.0%)	0 (0%)		
J2-064	309 (90.6%)	7 (2.27%)	1/2b	0
R2-502	297 (94.3%)	0 (0%)		
F2365	296 (94.6%)	1 (0.34%)	4b	0
ScottA	296 (94.6%)	0 (0%)		

**Table 7:** Enzymes catalyzing strain and serovar-specific essential reactions in simulations reflecting glucose minimal media (MM), queso fresco (QF), chicken breast (CB), smoked salmon (SS), cantaloupe (C), or romaine lettuce (RL).

Strain/Serovar	Unique Enzyme	MM	QF	CB	SS	C	RL
J2-031	S-Adenosyl-L-homocysteine hydrolase	+	+	+	+	+	+
	Oxalosuccinate:NADP+ oxidoreductase (decarboxylating)	-	+	-	-	-	-
	Isocitrate glyoxylate-lyase	+	+	+	+	+	+
	alpha-D-Glucose-1-phosphate:alpha-D-glucose-1-phosphate	+	+	+	+	+	+
	Xanthosine-5’-phosphate:L-glutamine amido-ligase (AMP-forming)	+	+	+	+	+	+
	Maltose:orthophosphate 1-beta-D-glucosyltransferase	+	+	+	+	-	+
	Adenosine:orthophosphate ribosyltransferase	+	+	+	+	+	+
	Isocitrate:NADP+ oxidoreductase (decarboxylating)	-	+	-	-	-	-
	beta-D-Glucose 1-phosphate 1,6-phosphomutase	+	+	+	+	-	+
J2-064	4-hydroxybenzaldehyde:NAD+ oxidoreductase	+	+	+	+	+	+
	ATP:thiamine phosphotransferase	+	+	+	+	+	+
	2-Methyl-4-amino-5-hydroxymethylpyrimidine-diphosphate:4-methyl-5-	-	+	+	+	+	+
	ATP:4-amino-2-methyl-5-phosphomethylpyrimidine phosphotransferase	-	+	+	+	+	+
	4-amino-2-methyl-5-phosphomethylpyrimidine synthetase	-	+	+	+	+	+
	thiazole phosphate synthesis	-	+	+	+	+	+
	4-hydroxy-benzyl-alcohol dehydrogenase	-	+	+	+	+	+
F2365	Adenosyl cobinamide kinase	+	+	+	+	+	+
1/2a	L-threonine ammonia-lyase_c0	+	-	-	-	-	-
	O-Acetyl-L-homoserine acetate-lyase (adding methanethiol)_c0	+	-	-	-	-	-
	2-Amino-4-hydroxy-6-hydroxymethyl-7,8-dihydropteridine:4-_c0	+	+	+	+	+	+

### Determination of defined minimal growth media

In order to utilize batch growth experimental data to quantitatively validate the GEMs created in this study, the media used to perform the batch growth experiments must be chemically defined so that the nutrient amounts can be accurately used for *in silico* growth simulations. Therefore, growth curves were generated to test several chemically defined media to determine which media would support growth of the six strains of *L*. *monocytogenes* for subsequent batch growth assays. IMM was the first medium tested, and growth was inconsistent across strains (S1A Fig). Strain ScottA was particularly problematic, but growth was only observed in strains J2-064 and JO161, with late growth in strain F2365. The second medium tested was MWB. Similar to IMM, growth was inconsistent (S1B Fig). Again, growth was not supported for strain ScottA, and strain F2365 was also not supported. However, growth was supported for the four remaining strains. Finally, MWB supplemented with 3% BHI was tested. This medium supported growth of all six strains (S1C Fig), and was; therefore, used for subsequent batch growth experiments.

### Conversion factors

The data generated by the batch growth experiments included data for viable cell counts, optical density, and biomass. Since this data was obtained for each of the six strains, it was possible to calculate conversion factors to convert one type of data to another. Calculated conversion factors were determined to compare and convert viable cell counts to biomass and optical density to biomass, which are the two most important conversions for subsequent model validation (Table 8).

**Table 8:** Experimentally determined conversion factors.

Strain	Viable Cells (CFU/mL) to Biomass (gDCW/L) ± SD^a^	OD_600_ to Biomass (gDCW/L) ± SD
J2-031	---^b^	1.27 x 10^−1^ ± 4.40 x 10^−2^
JO161	2.8 x 10^−10^ ± 3.3 x 10^−11^	3.78 x 10^−1^ ± 2.17 x 10^−1^
J2-064	4.4 x 10^−11^ ± 1.7 x 10^−11^	9.38 x 10^−2^ ± 4.79 x 10^−2^
R2-502	1.3 x 10^−10^ ± 6.4 x 10^−11^	2.27 x 10^−1^ ± 8.70 x 10^−2^
F2365	4.6 x 10^−11^ ± 1.2 x 10^−11^	8.19 x 10^−2^ ± 3.91 x 10^−2^
ScottA	4.3 x 10^−11^ ± 2.1 x 10^−11^	1.03 x 10^−1^ ± 7.60 x 10^−2^

^a^ Standard Deviation

^b^ Viable cell count was not taken for strain J2-031

### Batch growth and qualitative *in silico* refinement

Experimental growth rates (h^-1^) were calculated using linear regression on the four, hourly dry cell weight measurements taken for each strain. The initial *in silico* growth rate predictions were obtained by first using the optical density to biomass conversion factors (Table 8) to approximate the initial biomass at the start of each of the batch inoculum. The duration for completion of batch growth *in silico* was then adjusted to reflect the amount of time determined experimentally. The correlation between the experimental growth rates and *in silico* growth rate predictions was neither strong, nor significant (Pearson correlation test statistic yields p < 0.32). Therefore, the scalar differences between the experimental growth rates and the initial predictions were used to multiply the predicted biomass values in order to better reflect experimental growth rates. After introduction of the scalar factors determined for each strain, the correlation between the experimental growth rates and *in silico* predictions was much stronger and significant (p < 0.013). Similarly, the correlation between the experimental growth yields and the initial *in silico* predictions were neither strong, nor significant (p < 0.36). However, after using the scalar factors to adjust the models, the correlation became much stronger and significant (p < 0.0015).

**Fig 7:**
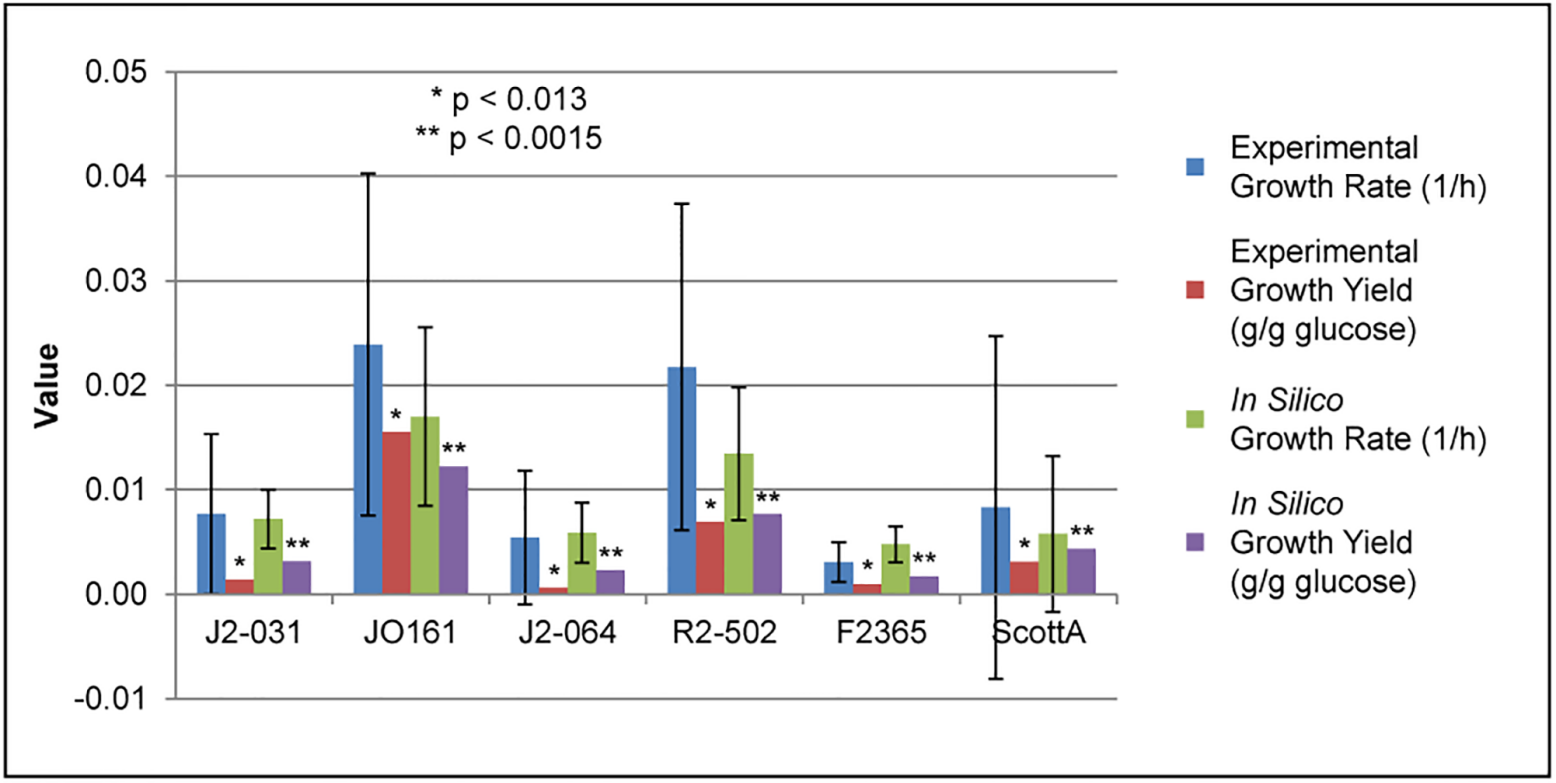
Comparison of experimental and *in silico* growth rates and growth yields including experimental standard deviations.

A graphical of representation of the growth characteristics obtained experimentally and predicted *in silico* was obtained (Fig 7). Data generated by the model before the scalar factors were introduced (data not shown), due to the stronger correlation between the experimental results and the predictions after the scalar factors were introduced.

## Discussion

When the content of predicted ORFs is compared, it is clear that much of the genome contents are shared by all six *L*. *monocytogenes* strains (Table 1). The genomes are all very similar in length, and greater than 85% of each genome is shared between all strains. This degree of conservation was expected, since most evidence suggests that the genomes of modern *L*. *monocytogenes* strains are highly conserved [24].

Interestingly, the amount of serovar specific genes is on the same scale as the amount of genes unique to each strain. This is represented by the fact that the number of serovar specific genes is similar to the number of unique genes (Table 1). The low frequency of serovar specific genes could possibly indicate that a new method of classification could be more representative of actual differences between strains.

As GEMs were curated and validated through comparison to experimental data, the number of genes, metabolites, and metabolic reactions for each of the GEMs at each stage of their development increased (Table 2). The models were very consistent in the number of genes, metabolites, and reactions. The vast majority of the metabolites (99.1%) and reactions (97.4%) were shared between all six of the GEMs. However, three of the strains (*L*. *monocytogenes* strains J2-031, J2-064, and F2365) had at least one unique reaction, as did serovar 1/2a. These unique reactions may have important implications, because they may give these strains a competitive advantage in specific environments, or may explain the isolation source of the strain.

The level of diversity in the metabolic genes and corresponding content was not too surprising. This is because *L*. *monocytogenes* is a non-spore forming bacteria, and similar studies with other non-sporulating bacteria that compare different strains of the same organism, such as *E*. *coli* or *Staphylococcus aureus*, saw similar levels of differentiation in metabolic gene/reaction content [1,2,9]. In terms of the numbers of these components (genes, reactions, and metabolites), the GEMs created in this study are very comparable to other GEMs not based on *E*. *coli* [4,6]. In contrast, when compared to models of *E*. *coli*, the models created in this study involve fewer genes, metabolites, and metabolic reactions, many of which are due to *E*. *coli* having an additional outer membrane and periplasm, which requires numerous additional transport and metabolic reactions for *in silico* inquiry [1,2]. In addition, *E*. *coli* K12 is the most studied bacterium in the world, and approximately 70% of functions of the genes in the genome have been experimentally characterized in the last century. In contrast, a paucity of research exists for the characterization of genes encoding metabolic enzymes and transporters specific to *L*. *monocytogenes*.

The nutrient utilization experimental data generated as part of this study is some of the first large-scale metabolic data for numerous strains of *L*. *monocytogenes* (S2 Dataset). The compounds uniquely utilized by each strain could give some insight on why that particular strain was isolated from its corresponding environment. For example, formic acid is a compound commonly used as a preservative in animal feed. Strain J2-031 was isolated from a bovine host [25], and was the only strain capable of metabolizing formic acid. It is possible that the utilization of formic acid as a carbon source from the animal’s feed provided a competitive advantage for J2-031 in a bovine host. Another example of unique nutrient metabolism is L-serine utilization as a carbon source by strain JO161. L-serine is a nonessential amino acid in humans, meaning it can be synthesized by the body. Since strain JO161 was isolated as a clinical sample from a human patient [25], it could be that utilization of excess L-serine may provide strain JO161 a competitive growth advantage in its host environment. Other examples are sucrose, fumaric acid, and tyramine as carbon sources for strain F2365. Sucrose is added to many foods, tyramine in naturally found in many foods, and fumaric acid is an approved food additive. Since strain F2365 was isolated from a food epidemic [25], utilization of one or more of these compounds could have contributed to its successful survival and proliferation.

The nutrient utilization data also shows trends of metabolic similarities between strains. For example, of the 95 carbon sources tested, 35 had identical utilization across all of the strains, but just 15 were unique to one strain, and only one was serovar specific. This means that 44 of the compounds providing sole sources of carbon were used by some combination of strains. Analysis of these combinations, while beyond the scope of this study, could provide valuable insight into the metabolism of *L*. *monocytogenes*. In contrast, the metabolism of nitrogen, phosphorus, and sulfur was much more consistent across all six strains. 89 of the 95 nitrogen sources had the same utilization among all strains, and 90 of the 94 phosphorus and sulfur sources were consistent across strains, thus supporting a high level of conservation in the metabolic capabilities for these nutrients.

Using phenotypic microarray plates to experimentally determine carbon source utilization phenotypic data is a very common method to validate GEMs for qualitative predictive accuracy. 57 of the carbon sources tested on Biolog^™^ PM1 plates were also present in the set of metabolites contained in the GEMs, thereby allowing comparisons of *in silico* and experimental nutrient utilization to be made. In the case of *E*. *coli* K12, multiple iterations have expanded the contents of the series of GEMs for this organism, and each iterative metabolic reconstruction has led to improvement of the accuracy of nutrient utilization predictions from 70% to 98% [1,16,26]. This indicates that the models created in this study are comparable to those already published, and, as such, they can be used in similar ways that these other models have been used, for example, simulating complex environments such as foods or host niches for determination of predicted essential reactions.

Nitrogen source utilization experiments are another common method used to assess the validity of GEMs for qualitative growth predictions. These results are not as promising as the carbon source utilization results; however, a more thorough review of the available genomic metabolism information could reveal possible opportunities for improvement since many of these nitrogen utilization pathways have not been extensively researched for *L*. *monocytogenes* strains. Because carbon source utilization is the most common form of validation of GEMs, the focus of this study was to focus on the accuracy of carbon utilization agreement, but nitrogen, phosphorus, and sulfur were also included to investigate how well GEMs created with semi-automated tools can predict utilization of these nutrients. Therefore, less attention was paid to manual curation to improve the predictive accuracy of *L*. *monocytogenes* GEMs for nitrogen metabolism, and these values could serve as a baseline for future studies to identify ways to improve genome-scale metabolic reconstruction pipelines.

In addition to carbon and nitrogen source utilization experiments, phosphorus and sulfur experiments are also performed and compared to *in silico* GEM predictions. Four previous GEMs were found that performed these experiments as part of the experimental validation process and their agreement can be seen in Fig 6. These GEMs had an average phosphorus agreement of 78.0 ± 42.0% and an average sulfur agreement of 45.3 ± 24.3%. In this study, there were 22 phosphorus and 11 sulfur sources for which comparisons were possible. However, a comparably low phosphorus agreement (15%) was seen in one of the studies, so low phosphorus agreement is not unique to this study, and is likely due to the paucity of experimental studies that focus to characterize genes and reactions involved in unique non-phosphate phosphorous sources. Conversely, the sulfur agreement is considerably better than that of the four comparison GEMs, (Fig 6). Much like the nitrogen metabolism, phosphorus and sulfur metabolism received less vigorous curation, and, while the sulfur agreement is promising, the phosphorus agreement could potentially be improved. Both phosphorus and sulfur agreements, like nitrogen, could serve as baselines for future work.

At the forefront of the possible applications for the use of GEMs is the identification of new methods for control for pathogens. This is primarily accomplished through the prediction of essential reactions. These metabolic reactions are those that are required for the organism to produce biomass and are likely necessary for viability and/or growth. They are identified by running *in silico* gene/reaction simulations: systematically simulating removal of one metabolic reaction at a time. These simulations attempt to optimize biomass production with the knocked out reaction. If no biomass is able to be produced, the reaction is considered essential. This approach has been successfully used with a GEM of *Vibrio vulnificus* to identify new control targets, and through screening of metabolite analog libraries of compounds, led to new ways to kill and prevent the growth of *V*. *vulnificus* [6].

In this study, essential reaction simulations were first determined using the final, manually curated and validated GEMs in conditions simulating a glucose minimal media. Then, chemical compounds present the analytical composition of five of the foods most frequently recalled for *L*. *monocytogenes* contamination were added to the simulation medium, and essential reaction predictions were determined for each of these simulated environments. The five foods chosen were queso fresco, chicken breast, smoked salmon, cantaloupe, and romaine lettuce.

Summaries of the essential reaction predictions can be seen in Table 8. Simulations run on the glucose minimal media predicted the most essential reactions (average = 368.3 ± 1.5). This was expected, since the food matrices that were simulated had additional nutrients available, which would allow the organism to compensate with alternative catabolic methods when certain metabolic reactions were eliminated. All of the essential reaction simulations done on foods predicted similar numbers of essential reactions (average = 298.3 ± 4.8). This was also expected, because the models did not contain all of the different nutrients available in each of the different foods, and; therefore, the compositions of the simulated media were very similar.

The predictions of essential reactions generated by this study can be used by future studies to identify new methods of control for a broad spectrum of *L*. *monocytogenes* strains. The logical next step from this analysis is for future studies to experimentally validate the essentiality of the metabolic reactions. This can be accomplished by performing gene knockout experiments, similar to previous studies which combined mutant library screening and metabolic modeling to identify new roles of genes important for *L*. *monocytogenes* strain EGD-e during intracellular replication [10]. Knocking out, or preventing the expression of, the genes associated with the predicted essential reactions should be lethal to the organism. Once the essentiality of the reactions has been confirmed, new drugs, such as chemical analogs that mimic metabolites that irreversibly bind to the essential enzyme, or other control methods, can be developed to target the genes and their associated reactions. There has been success for using GEM essential reaction predictions to aid in the development of drugs targeting foodborne pathogens, specifically *Vibrio vulnificus* [6].

The importance of running qualitative batch growth experiments on chemically defined media cannot be understated. Without a precise, known nutrient composition, the environmental conditions used for the growth simulations will not be accurate, which undermines the accuracy with which the GEMs can make accurate predictions. Difficulties arose in achieving consistent growth on both IMM and MWB. Multiple trials were run for each media, but little consistency across strains and the inability to reproduce the work of Phan-Thanh *et al*. [27] and Premaratne *et al*. [28] resulted in the decision to supplement MWB media with 3% BHI. This is likely due to minute requirements of vitamins or cofactors absent in the previously described minimal media recipes. While this slightly compromises the chemical composition of the media, it resulted in consistent overnight growth in the media, which made the qualitative batch growth experiments feasible.

Some degree of variation in the conversion factors was observed (Table 8). One potential reason for the uncertainty is the relatively few measurements taken of dry cell weight (4) and viable cell concentration (1). While these measurements, taken in triplicate, allow for the generation of necessary growth rates and growth yields, the values for determination of conversion factors may be improved by increasing the number of time points used. However, the values used here to determine *in silico* growth rates and growth yields had a strong correlation to experimental values and were determined to be statistically significant. Therefore, these values provide new tools for the field of systems biology and for future studies performing *in silico* work with *L*. *monocytogenes* strains.

When comparing the conversion factors generated in this study to those previously published [1], it can be seen that the optical density to viable cell concentration factors are on similar scales (average = 0.168 and 0.418 for *L*. *monocytogenes* and *E*. *coli*, respectively). This was expected, since it is a comparison of two bacteria. Conversely, the conversion factors for viable cells to biomass differ by several orders of magnitude (average = 1.09 x 10^−10^ and 3.27 x 10^−7^ for *L*. *monocytogenes* and *E*. *coli*, respectively). The degree to which the viable cells to biomass conversion factors differ is not surprising, since *E*. *coli* is Gram negative and *L*. *monocytogenes* is Gram positive and the additional outer membrane and periplasm found in Gram negative bacteria may account for some of these differences.

Batch growth experiments were conducted in order to make several comparisons between six *L*. *monocytogenes* strains representing the three serovars (1/2a, 1/2b, and 4b) used in this study. The first comparison made was of experimental growth rates and growth yields between the six strains representing the three serovars. It was determined that there was no statistically significant (student’s t-test statistic yields p > 0.16) difference in growth rate between any of the strains or any of the serovars. A trend was observed in which serovar 4b had a lower growth rate (average = 0.00569 h^-1^) than either serovar 1/2a (0.01580 h^-1^) or 1/2b (0.01359 h^-1^), but the difference was not significant (p > 0.42). Similarly, there was no significant difference observed (p > 0.13) in growth yields between strains or serovars. As with growth rates, there was a trend that indicated serovar 4b had lower growth yields (average = 0.00528 g/g glucose) than either serovar 1/2a (0.01210 gDCW/g glucose) or 1/2b (0.00966 gDCW/g glucose), but again, the difference was not significant (p > 0.40).

The second comparison was of the initial *in silico* predictions of growth rates and growth yields between serovars. As was seen with the experimental values, there was no significant (p > 0.06) difference between any of the serovars for either growth rate or growth yield. Again reflecting the experimental values, an observed trend indicating serovar 4b had a lower growth rate (average = 0.02857 h^-1^) and growth yield (average = 0.01270 gDCW/g glucose) than either serovar 1/2a (0.05835 h^-1^ and 0.02594 gDCW/g glucose) or 1/2b (0.07500 h^-1^ and 0.03333 gDCW/g glucose). However, neither of these trends were significant (p > 0.06).

Next, we sought to compare the experimental growth rates and growth yields and the initial *in silico* predictions of these values. This was in order to see how close the initial *in silico* predictions were to the experimental results. This comparison revealed that the initial predictions were considerably inaccurate. Initial growth rate predictions were all at least one standard deviation above the experimental average, and for several of the strains, the predictions were much more than one standard deviation above. Similarly, all of the initial growth yield predictions were at least one standard deviation above the experimental average, except for strain JO161, which was just inside one standard deviation. These results, while good in that all strains were consistently over-estimated, indicated that steps needed to be taken to improve accuracy of quantitative *in silico* growth predictions, namely the introduction of scalar factors similar to the work with *E*. *coli* K12 GEMs [29].

Next, we sought to determine differences through comparison of the *in silico* predictions for growth rate and growth yield after the introduction of the scalar factors. Similar to the experimental and initial model comparisons, there was no significant (p > 0.48) difference between serovars. The trends indicating that serovar 4b had the lowest growth rate (average = 0.00205 h^-1^ vs. 0.00848 and 0.00379 h^-1^) and growth yield (average = 0.00302 gDCW/g glucose vs. 0.00768 and 0.00498 gDCW/g glucose) were less pronounced after the introduction of the scaling factors. Therefore, it was not unexpected that the trends, like those for experimental and initial model comparisons, were not significant (p > 0.48).

To improve quantitative *in silico* predictions, we compared the experimental and *in silico* values for growth rate and growth yield after the introduction of scalar factors into the FBA algorithm. As shown, the determination and implementation of the scalar factor greatly improved the accuracy of the *in silico* predictions (Fig 7). With the use of the scaling factors to multiply the biomass value, all of the *in silico* prediction values for growth yields correlated with the experimentally determined values, and all fell within one standard deviation from the average. Similarly, all but one of the *in silico* prediction values for growth rates correlated with the experimentally determined values, and fell within one standard deviation from the average. The one exception was strain F2365, which was just outside of one standard deviation from the experimental average. These results, combined with the strong, significant (Pearson correlation test statistic yields p < 0.013 and p < 0.0015) correlation between the experimental values, indicate that using the scalar factors was a valid method for improving the predictive accuracy of the GEMs. This is similar to the work conducted with various iterations of *E*. *coli* K12 GEMs, which has been useful for bioengineering strains for production of commodities and examining the evolutionary relationships of numerous pathogenic and nonpathogenic *E*. *coli* strains [1,16,26]

## Conclusions

This study describes the creation, multi strain comparison, and qualitative experimental validation of six GEMs for *L*. *monocytogenes*. Nutrient utilization comparisons were made for sole sources of carbon, nitrogen, phosphorous, and sulfur. The models were then curated to better agree with the experimental results for carbon utilization, since that is the method most commonly used to validate GEMs. It was found that the final carbon source utilization agreement of the newly created models was comparable to the agreement seen by previously published models. Additionally, the nitrogen and phosphorus agreements were lower than previously published models, but the sulfur agreement was higher than previously published models. Quantitatively validating the GEMs as laboratory tools was done using batch growth experiments, and determination of scalar factor adjustments resulted in growth rate and growth yield predictions that were much more accurate, and were significantly correlated with the experimental results. The potential essential reactions identified by this study provide promising directions for future studies improving the safety of foods commonly contaminated by *L*. *monocytogenes*, including cheese, deli meats, salads, and fruit. The models could also be used for future iterations of GEMs for *L*. *monocytogenes*. These results indicate that GEMs generated by semi-automated tools provided by KBase, followed by manual curation and experimental validation, can also become successful predictive tools for use in the laboratory.

## Materials and methods

### Stock culture maintenance

The six strains chosen for this study represent a variety of isolation sources as well as each of the three most prevalent serovars in terms of human listeriosis cases (Table 1). Culture stocks were kept at -80°C for long term storage. Strains were streaked onto brain heart infusion (BHI) agar plates (Becton, Dickinson, and Company, Sparks, MD) and stored at 4°C. The refrigerated cultures were re-streaked onto fresh BHI agar plates every two weeks as needed.

### Generation of genome-scale metabolic models

Six *Listeria monocytogenes* strain genomes (F2365, J2-031, J2-064, JO161, R2-502, and Scott A) were downloaded in GenBank genome files obtained from the National Center for Biotechnology Information (NCBI) database [30,31,32,33,34]. These genomes were then uploaded to KBase, an online genome-scale metabolic reconstruction pipeline sponsored by the U.S. Department of Energy, and draft GEMs were created using the semi-automated tools provided by KBase [Department of Energy Systems Biology Knowledgebase (KBase), http://kbase.us] that is built with the same bioinformatics pipeline as the ModelSeed [35]. In the first step of the Model SEED or K-Base pipeline, the assembled genome sequence is annotated by the Rapid Annotation using Subsystem Technology (RAST) server [15] and imported into the SEED analysis system. Next, a preliminary GEM is generated consisting of intracellular and transport reactions associated with genes on the basis of RAST annotations, spontaneous reactions and an organism-specific biomass reaction. In the gap-filling step of the pipeline, additional intracellular and transport reactions are added to create an analysis-ready GEM capable of simulating biomass production using only transportable nutrients.

To make GEMs functional, it is a common practice to identify missing reactions required for metabolic pathways that are known to be functional based on experimental evidence, but the gene encoding the reaction may have not yet been identified. This gap-filling process was required in this study to fill in missing metabolic reactions required for the utilization of D-glucose, a compound that is known to be able to be metabolized by *L*. *monocytogenes* and nearly all free living bacteria. Therefore, using KBase, gap-filling was performed on the draft *L*. *monocytogenes* models for D-glucose under both aerobic and anaerobic conditions. Then the draft models were downloaded in systems biology markup language (SBML) file format, curated through comparison to experimental data, and provided here as validated SBML Level 3 fbc format files (S1, S2, S3, S4, S5 and S6 Files) with provision of upper and lower bounds used for Flux Balance Analysis (FBA) simulations (S1 Dataset). The high level modeling system for mathematical optimization GAMS (General Algebraic Modeling Software) and CPLEX solver was used to conduct computational analysis using established methods such as Flux Balance Analysis, dynamic FBA, and Gene/Reaction essentiality predictions from the COBRA toolbox [36]. Cellular growth rate (or biomass production) is often used as an objective function for FBA, and was used for FBA analyses performed in this study. The biomass equations for each model were identical as provided by K-Base and are based on the analytically determined amount of metabolites from the *E*. *coli* biomass equation to comprise 1 g of cellular dry cell weight [16,37], and growth and non-growth associated ATP requirement values, and PO (number of ATP molecules produced per pair of electrons donated to the electron transport system) ratio were used for all models as previously described [16].

### *In vitro* nutrient utilization

Biolog^™^ PM1, PM3, and PM4 plates (Biolog, Inc., Hayward, CA) were used for all *in vitro* nutrient utilization experiments. When preparing Biolog^™^ plates, three isolated colonies from each refrigerated culture plate were inoculated into separate test tubes containing 9 mL of BHI broth. The BHI tubes were then incubated at 37°C overnight. After incubation, two tubes for each strain were chosen and 300 μL from each tube were taken and inoculated onto separate Biolog Universal Growth plus sheep’s Blood (BUG+B) agar plates in triplicate (6 plates per strain). The BUG+B plates were then incubated at 37°C overnight. After incubation, one BUG+B plate was harvested using a sterile cotton swab, and cells were placed in 15 mL conical tubes containing 1.8 mL of IF-0 solution, prepared as directed by the manufacturer. 150 μL of cell suspension was removed, placed in a new 15 mL conical tube, and the OD_590_ was then adjusted to 0.171 ± 0.020 by diluting with fresh IF-0 solution. After OD_590_ adjustment, 1.8 mL of the diluted cell suspension was added to 15 mL conical tubes containing 9 mL of IF-0+ solution that had been prepared according to manufacturer instructions. For PM3 and PM4 plates, 108 μL of 100X ferric citrate/sodium succinate solution was added to each conical tube containing cellular suspension. The resulting solution was then transferred to a sterile reservoir, and then transferred to separate wells of Biolog^™^ plates, separate plates for each replicate of each strain (12 total), in 100 μL aliquots. 640 nm absorbance values were then taken at 0, 12, 24, 48, and 72 hour time points using a ChroMate^®^ Microplate Reader (Awareness Technology, Inc., Palm City, FL).

### *In silico* nutrient utilization prediction and reconciliation

GAMS is an optimization software package and was used to perform flux balance analysis (FBA) on each of the models to determine predicted nutrient utilization capabilities using methods previously described [36]. FBA is a technique frequently used while studying GEMs. This is because FBA makes growth rate predictions possible by calculating the flow of metabolites through the network [38]. The foundation of FBA is the translation of a metabolic network into a matrix of stoichiometric coefficients, the S matrix. This matrix includes each metabolite in the network as a separate row and each metabolic reaction as a separate column. Representing the metabolic network in this fashion places constraints on the system in the form of a steady state mass balance, that is, the rate of consumption of metabolites cannot exceed the rate of production. Additional constraints, such as maximum or minimum values for reaction fluxes, can also be implemented [38]. The function of these constraints is to restrict the possible range of reaction fluxes into a defined solution space. Growth rate predictions are enabled by representing the production of biomass as a metabolic reaction, which serves as the objective function, and adds a column to the S matrix. This allows the optimization of the objective function by calculating the flux distribution that results in the maximum flux through the biomass reaction [38]. In terms of nutrient utilization, when the optimized biomass reaction results in zero flux, it is determined that the organism cannot utilize that nutrient as a sole source of carbon, nitrogen, phosphorous, or sulfur.

After the initial nutrient utilization predictions, the metabolites contained in the GEMs were surveyed to identify compounds matching those present on Biolog^™^ plates that did not already have corresponding transport reactions in the draft GEMs. After identifying the missing compounds, the models were manually curated to add the required reactions in order to generate predictions for *in silico* nutrient utilization. These predictions were then compared to *in vitro* nutrient utilization experiments using Biolog^™^ plates.

Comparisons of *in silico* predictions to *in vitro* results generated a list of disagreements necessary to reconcile for validation of GEMs predictive accuracy. The GEMs were then manually curated to remove transport reactions associated with compounds for which *in vitro* results showed no growth but *in silico* predictions predicted growth (false positives). Compounds for which *in silico* simulations predicted no growth, but *in vitro* experiments showed growth (false negatives) were reconciled by first using KBase to gapfill the draft models on those compounds to identify any necessary missing metabolic reactions. The reactions in the resultant secondary draft models were then used as templates by which the manually curated models could be updated. This step was necessary because the manually curated models could not be re-uploaded to KBase and repeatedly gapfilled. The gapfilling, manual curation, and Biolog^™^ experimental validation techniques presented here are common methods used to validate and optimize GEMs [1,37,39,40].

### Essential reaction predictions

Essential reactions are those required by a microorganism for viability and/or growth in a given environmental condition. Therefore, determination of essential reactions identifies metabolic reactions and corresponding genes as ideal targets for control of viability and growth. Predictions of essential reactions can be accomplished *in silico* by adding additional constraints to the FBA simulations of GEMs. The constraints introduced during essential reaction prediction simulations serve to restrict the flux through each metabolic reaction one at a time. FBA then re-optimizes the objective function (biomass production) while the single reaction is restricted. If, lacking the specific metabolic reaction, the predicted biomass is zero, the restricted reaction is deemed essential. This approach for predicting essential reactions with GEMs is well established [1,41]. Using a similar approach, where essential metabolites were predicted Kim *et al*. were able to identify successful metabolite-mimicking drugs that had a bactericidal effect on the opportunistic foodborne pathogen, *Vibrio vulnificus* [6].

Essential reaction predictions were performed after final reconciliation between *in silico* predictions and *in vitro* experimental results. GAMS was used to simulate gene knockouts, eliminating one metabolic reaction at a time by constraining the flux to zero in order to identify which of those reactions were essential to the survivability and viability of each of the six strains of *L*. *monocytogenes*. Essential reactions were determined by a prediction of zero biomass generation associated with the removal of the reaction. Essential reaction predictions were first performed in conditions reflecting the nutrients present in a minimal media with glucose as the sole energy source (H_2_O, PO_4_^3-^, CO_2_, NH_3_, Mn^2+^, Zn^2+^, SO_4_^2-^, Cu^2+^, Ca^2+^, H^+^, Cl^-^, Co^2+^, K^+^, Mg, cob(I)alamin, Na^+^, Fe^2+^, Fe^3+^, O_2_, and D-glucose). Subsequently, the chemical composition of five of the foods most commonly recalled for *L*. *monocytogenes* contamination was obtained from the United States Department of Agriculture (USDA) nutrient database. Using this chemical composition, compounds that were both present in the foods and present in the models were added to the environmental simulations in reported analytical amounts (S1 Dataset) to see if any differences in essential reaction predictions arose when growth was simulated on each food item. The five food items for which essential reaction predictions were generated were queso fresco, chicken breast, smoked salmon, cantaloupe, and romaine lettuce (Table 9).

**Table 9:** Nutrients added to essential reaction simulations to reflect high-risk foods.

Nutrient	Queso Fresco	Chicken Breast	Smoked Salmon	Cantaloupe	Romaine Lettuce
L-Glutamate	+	+	+	+	+
Glycine	+	+	+	+	+
L-Lysine	+	+	+	+	+
L-Aspartate	+	+	+	+	+
L-Arginine	+	+	+	+	+
L-Glutamine	+	-	-	-	-
L-Serine	+	+	+	+	+
L-Methionine	+	+	+	+	+
L-Tryptophan	+	+	+	+	+
L-Phenylalanine	+	+	+	+	+
L-Tyrosine	+	+	+	+	+
Sucrose	-	-	-	+	-
D-Fructose	-	-	-	+	+
L-Cysteine	+	+	+	+	+
Choline	+	+	+	+	+
L-Leucine	+	+	+	+	+
D-Alanine	+	+	+	+	+
L-Histidine	+	+	+	+	+
L-Proline	+	+	+	+	+
L-Asparagine	+	-	-	-	-
L-Valine	+	+	+	+	+
L-Threonine	+	+	+	+	+
Maltose	-	-	-	+	-
Palmitate	+	+	+	+	+
Riboflavin	+	+	+	+	+
Thiamin	+	+	+	+	+
L-Isoleucine	+	+	+	+	+
Vitamin B12	+	+	+	-	-

### Growth assays

The strains of *L*. *monocytogenes* used for quantitative validation are the same six strains used for qualitative validation (Table 1). To ensure the successful growth of these strains of *L*. *monocytogenes* in the selected minimal media, growth curves were generated using 96 well plates and the BioTek^®^ Epoch 2 Microplate automated spectrophotometer (BioTek Instruments, Inc., Winooski, VT). Three chemically defined media were tested. The first was an improved minimal medium (IMM) described by Phan-Thanh *et al*. [27]. The second was Modified Welshimer’s Broth (MWB), as described by Premaratne *et al*. [28]. The final, and most successful, medium tested was MWB supplemented with 3% BHI broth. When generating a growth curve, four isolated colonies were inoculated into separate test tubes filled with 9 mL of the chosen chemically defined medium. These tubes were then incubated at 37°C for 24 hours with shaking at 250 rpm. After incubation, three of the samples for each strain (18 total) were transferred to sterile 15 mL conical tubes, and the OD_600_ was adjusted to 0.040 ± 0.010 using fresh media. After the OD_600_ adjustment, 200 μL of each sample was placed into separate wells of a 96 well plate, along with triplicate wells of fresh, uninoculated media as the negative growth control. The 96 well plate was then placed in the plate reader, where OD_600_ measurements were taken every 10 minutes for 48 hours at 37°C. Every six readings were then averaged to generate a growth curve with hourly time points. Inconsistent growth was observed with the first two chemically defined media tested (IMM and MWB), but growth was supported by MWB supplemented with 3% BHI.

### Batch growth experiments

In order to grow *L*. *monocytogenes* in sufficient quantities to perform dry cell weight measurements, batch spargers, as described by Sutton *et al*. [42], were used. The day prior to the experiment, MWB supplemented with 3% BHI broth (MWB3) was prepared and pipetted in 15 mL aliquots into nine sterile test tubes. These tubes were then each inoculated with an isolated colony of the *L*. *monocytogenes* strain to be tested. The tubes were incubated overnight at 37°C with shaking at 250 rpm.

MWB3 was prepared fresh prior to the start of each experiment and 600 mL aliquots were placed into three separate, sterile 1 L glass sparger bottles. Additionally, 50 mL were prepared for use as spectrophotometer blanks. A 100 μL sample of the overnight culture was taken and diluted so that the OD_600_ was 0.040 ± 0.010. Then, the remaining overnight culture was used to inoculate the three batches of 600 mL MWB3 such that the dilution was equivalent to the dilution required to obtain the desired OD_600_ in the 100 μL sample. A sample from each of the batch spargers was then taken, and its OD_600_ measured. Subsequent OD_600_ measurements were taken hourly for each of the batches for the duration of the run.

### Dry cell weight measurements

Dry cell weight measurements were performed in conjunction with the aerobic batch growth experiments. 12 (3 batches x 4 time points) Whatman^™^ glass microfiber filters (GE Healthcare Bio-Sciences, Pittsburgh, PA) were labeled and placed in an 80°C oven and left overnight to dry. One hour prior to beginning dry cell weight experiments, the dry filter weight was measured and recorded as described by Baumler *et al*. [1]. The first time point for the dry cell weight experiment was when the OD_600_ of the batch growth spargers reached 0.100.

When the OD_600_ reached 0.100, approximately 60 mL was extracted from each batch. 50 mL of each sample was vacuum filtered through its corresponding pre-weighed filter paper. After the culture was filtered, the paper was washed with approximately 5 mL of sterile DI water, left to dry for two minutes, and washed again with approximately 5 mL of sterile DI water. When all three of the filter papers for the time point were finished, they were placed in an 80°C oven to dry for a minimum of 24 hours. This process was repeated hourly for a total of four time points. 24 hours after the last time point, the filters were re-weighed, and the dry cell weight was determined from the difference between the pre-weighed filter mass, and the post-filtration mass.

### Viable cell counts

At the second dry cell weight time point, a CFU/mL count was determined for each of the three batches. Duplicate samples from each batch were taken and serially diluted to 10^−9^. The six highest dilutions (10^−4^–10^−9^) were then plated on BHI agar plates, incubated at 37°C for 24 hours, and enumerated.

### *In silico* quantitative adjustment

Following the completion of the batch growth dry cell weight experiments, the experimental data was used to adjust the models and improve the accuracy of the *in silico* predictions for biomass corresponding to growth rate. First, the data was used to generate conversion factors between biomass and optical density and between biomass and viable cell counts. Subsequent analysis of the data generated experimental growth rates (h^-1^), which were compared to *in silico* predicted growth rates to generate a scaling factor as previously described for the *E*. *coli* K12 GEMs [29]. This scaling factor was then used as a multiplier of biomass for FBA and dynamic FBA to improve the accuracy of *in silico* growth predictions. Initial *in silico* growth rates were determined by adjusting *in silico* glucose uptake rates until the duration of the batch growth matched experimental values. These glucose uptake rates were then held constant when the scaling factor was introduced, allowing the scaling factor to increase the accuracy of predictions of biomass used to generate values for growth rate and growth yield.

### Statistical analysis

Student’s t-test and Pearson correlation statistics in this study were conducted using Microsoft Excel.

## Supporting information

S1 File Computational model 1. SBML format of the final model of *L*. *monocytogenes* strain J2-031 for distribution and use in other modeling environments.
(XML)

S2 File Computational model 2. SBML format of the final model of *L*. *monocytogenes* strain JO161 for distribution and use in other modeling environments.
(XML)

S3 File Computational model 3. SBML format of the final model of *L*. *monocytogenes* strain J2-064 for distribution and use in other modeling environments.
(XML)

S4 File Computational model 4. SBML format of the final model of *L*. *monocytogenes* strain R2-502 for distribution and use in other modeling environments.
(XML)

S5 File Computational model 5. SBML format of the final model of *L*. *monocytogenes* strain F2365 for distribution and use in other modeling environments.
(XML)

S6 File Computational model 6. SBML format of the final model of *L*. *monocytogenes* strain ScottA for distribution and use in other modeling environments.
(XML)

S1 Dataset Nutrients taken up and secreted for FBA simulations of minimal growth medium, and foods used in this study. (XLSX)

S2 Dataset Experimental carbon, nitrogen, phosphorus, and sulfur utilization data for the six *L*. *monocytogenes* strains examined in this study. (XLSX)

S3 Dataset *In silico* predictions of nutrient utilization generated by the final, curated versions of the six newly created genome-scale metabolic models in this study. (XLSX)

S4 Dataset List of reactions added to the draft versions of the six genome-scale metabolic models created during this study. (XLSX)

S5 Dataset Experimental data for viable cell counts, dry cell weight, and optical density for all six strains examined in this study. (XLSX)

S1 Fig Microwell growth assay of six *L*. *monocytogenes* strains in A) IMM, B) MWB, and C) MWB supplemented with 3% BHI at 37°C including standard deviations. (DOCX)
