# Supplementary material for: Using genome-scale metabolic models to compare serovars of the foodborne pathogen Listeria monocytogenes
Source: PLoS One. 2018 Jun 7;13(6):e0198584. doi: 10.1371/journal.pone.0198584 (PMC6012718; doi:10.1371/journal.pone.0198584)
Supplement: S1 Fig — (DOCX) [file pone.0198584.s012.docx]

**Supplemental File 9: Figure S1.**

**A**

**B**

**C**
